# Advances in Multi-Omics Study of Prognostic Biomarkers of Diffuse Large B-Cell Lymphoma

**DOI:** 10.7150/ijbs.67892

**Published:** 2022-01-09

**Authors:** Xiao-jie Liang, Xin-yu Song, Jia-lin Wu, Dan Liu, Bing-yu Lin, Hong-sheng Zhou, Liang Wang

**Affiliations:** 1 Department of Hematology, Nanfang Hospital, Southern Medical University, Guangzhou 510515, China.; 2 The First Clinical Medical College, Guangdong Medical University, Zhanjiang 524000, China.; 3 Department of Interventional Radiology, The Third Affiliated Hospital of Sun Yat-sen University, Guangzhou 510630, China.; 4 Department of Hematology, Beijing Tongren Hospital, Capital Medical University, Beijing 100730, China.

**Keywords:** Diffuse large B cell lymphoma, Prognosis, Precision medicine, Systems biology, Biomarkers

## Abstract

As the most common subtype of non-Hodgkin's lymphoma, diffuse large B-cell lymphoma (DLBCL) is characterized by a huge degree of clinical and prognostic heterogeneity. Currently, there is an urgent need for highly specific and sensitive biomarkers to predict the therapeutic response of DLBCL and assess which patients can benefit from systemic chemotherapy to help develop more precise therapeutic regimens for DLBCL. Systems biology (holistic study of diseases) is more comprehensive in quantifying and identifying biomarkers, helps addressing major biological problems, and possesses high accuracy and sensitivity. In this article, we provide an overview of research advances in DLBCL prognostic biomarkers made using the multi-omics approach of genomics, transcriptomics, epigenetics, proteomics, metabonomics, radiomics, and the currently developing single-cell technologies.

## Introduction

Diffuse large B cell lymphoma (DLBCL) is the most common malignancy of the lymphohematopoietic system in adults, accounting for approximately 35% of non-Hodgkin's lymphomas 1 and has an aggressive clinicopathological course 2. In recent years, there have been significant improvements in the diagnosis and therapy of DLBCL. Its treatment is mainly immunochemotherapy-based. Forty to fifty percent of the DLBCL patients remain incurable after first-line treatment with rituximab, cyclophosphamide, adriamycin, vincristine, and prednisone (R-CHOP) 3. Therefore, highly specific and sensitive prognostic biomarkers are needed for exploring novel therapeutic targets, developing personalized therapies and post-treatment monitoring of DLBCL patients, ultimately improving their survival rate. Additionally, the heterogeneity and high recurrence rate of DLBCL makes early prognostic biomarkers essential for the development of treatment regimens and prognosis assessment.

**Figure 1:**
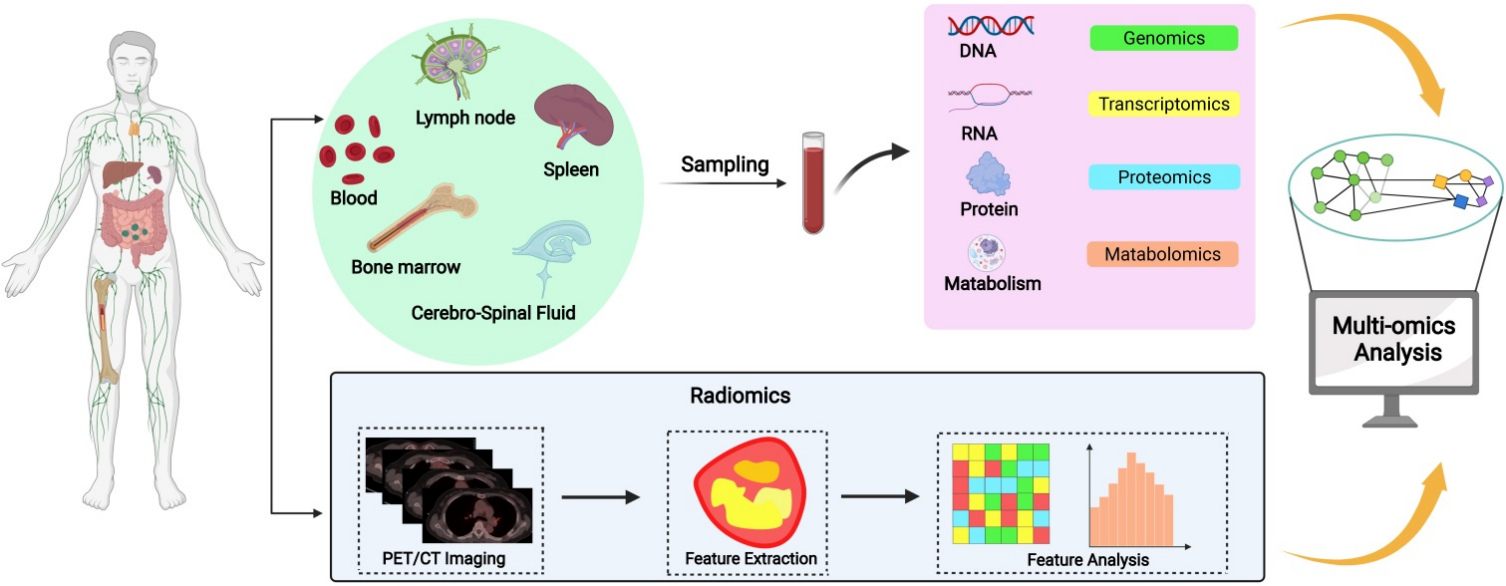
**A systematic review of prognostic biomarkers of DLBCL.** Diverse sample sources including lymph nodes, spleen, bone marrow, blood and cerebrospinal fluid provide diverse options for the screening of DLBCL biomarkers. Systems biology integrates data from multi-omics studies and uses multiple methods to analyze and process the data to gain a comprehensive understanding of the molecular mechanisms of DLBCL and identify prognostic biomarkers, but these biomarkers must be validated through clinical trials before clinical application.

Recent studies have shown that there are differences in predicting the prognosis of DLBCL patients based on cell origin classification. In the last decade, the advent of high-throughput genomic sequencing platforms, particularly whole-exome sequencing has helped identifying many genetic alterations unique to DLBCL 4. Systems biology approach, which allows researchers to observe and solve medical problems from a holistic perspective by combining data from genomics, transcriptomics, epigenetics, proteomics, metabolomics, and imaging, may be better suited to identify prognostic biomarkers for DLBCL. In this review, we have systematically reviewed the various multi-omics researches on DLBCL prognostic biomarkers (**Figure 1**), aiming to promote the precision medicine in DLBCL.

## Biomarkers of DLBCL subtypes

In 2000, Alizadeh et al. classified DLBCL into two subtypes based on its gene expression profile obtained using DNA microarray: germinal center B cells (GCB) and activated B cells (ABC) 5. They suggested that the overall survival (OS) of patients with the GCB subtype was significantly longer than that of patients with the ABC subtype. Subsequently, in 2002, Rosenwald et al. used DNA microarrays to determine gene expression in 240 DLBCL biopsy samples and identified three gene expression subgroups of DLBCL: GCB-like, ABC-like, and unclassified DLBCL. Since the survival curves of ABC-like DLBCL and unclassified DLBCL are inseparable, they are now collectively referred as non-germinal center B cell-like (non-GCB) 6. Although accurate, these molecular subtyping are costly and complicated, making their clinical implementation difficult. Therefore, Hans et al. proposed immunohistochemistry (IHC) as an alternative to microarray testing. They found that the presence of three proteins, CD10, BCL-6, and MUM1, could be used as a basis for DLBCL subtyping. DLBCL subtyping and survival analysis based on its IHC profile did not differ significantly from molecular subtyping 7. However, about 20% disconcordance may occur between Hans's algorithm and genomic subtyping 8. Thus, genomic analysis should be done to confirm the subtyping when it is highly suspected that the clinical findings do not match the Hans subtyping.

Molecular variant landscape of DLBCL has improved with the development of the next-generation sequencing technology (NGS). The increasingly accurate NGS-based molecular subtyping of DLBCL has greatly improved the prognostic assessment of patients. Schmitz et al. carried out exome and transcriptome sequencing on 574 DLBCL biopsy samples. They conducted DNA copy number analysis using chip technology, and targeted amplicon resequencing of 372 genes. Consequently, they identified four prominent genetic subtypes in DLBCL, termed MCD (based on the co-occurrence of MYD88^L265P^ and CD79B mutations), BN2 (based on BCL6 fusions and NOTCH2 mutations), N1 (based on NOTCH1 mutations), and EZB (based on EZH2 mutations and BCL2 translocations). This tetrad subtyping system is valuable in predicting outcomes, with BN2 and EZB subtypes having better survival than that of MCD and N1 subtypes 9. Increasing studies have confirmed the efficacy of BTK inhibitors in MCD subtype, especially patients with primary central nervous system DLBCL, 37% of whom are classified as MCD subtype 10. However, 53.4% of the patients could not be typed using this method. Using data from 304 DLBCL patients, Chapuy et al. performed a multilevel genetic analysis pertaining to low-frequency alterations, captured recurrent mutations, somatic copy number alterations (SCNAs), and structural variants (SVs). They used a consistent clustering method for integration to identify five DLBCL subtypes: 1) Cluster 1 (C1), 2) Cluster 2 (C2), 3) Cluster 3 (C3), 4) Cluster 4 (C4) and, 5) Cluster 5 (C5). The C1 subtype is a previously unidentified group of low-risk ABC-DLBCLs; the C2 subtype often exhibits 9p21.13/CDKN2A and 13q14.2/RB1 copy deletions; 95% of C3 subtypes are of GCB origin and exhibit BCL2, KMT2D, CREBBP, and EZH2 mutations, PTEN inactivation and epigenetic enzyme alterations; C4 subtypes are a newly defined group of high-risk GCB-DLBCLs; and the C5 subtype exhibits increased 18q copy number and high BCL2 expression. Although both C4 and C3 are predominantly GCB types, unlike the PETN alterations in C3, they are more common in C4 RHOA mutations 1. In 2020, Stuart et al. analyzed the mutation characteristics of a population-based cohort of 928 DLBCL patients by targeted sequencing of 293 genes and applied Bernoulli mixture-model clustering to classify patients into five molecular subtypes: 1) MYD88, 2) BCL2, 3) SOCS1/SGK1, 4) TET2/SGK1, and 5) NOTCH2, along with a “not elsewhere classified (NEC)” group. The BCL2 subtype, SOCS1/SGK1 subtype, and TET2/SGK1 subtype had good prognosis, with 5-year OS rates of 62.5%, 64.9%, and 60.1%, respectively; the MYD88 subtype had the worst prognosis, with a 5-year OS of 42.1%; NOTCH2 subtype and NEC subtype had intermediate prognosis with 5-year OS of 48.1% and 53.6%, respectively 11.

Wright et al. subtyped tumors based on the genetic profile of DLBCL patients. They created the LymphGen algorithm to provide a probabilistic classification of tumors that translate from individual patients to genetic subtypes, allowing for the classification of DLBCL into seven genetic subtypes: 1) MCD, 2) N1, 3) A53, 4) BN2, 5) ST2, 6) EZB-MYC+, and 7) EZB-MYC-, with a patient prevalence of 8.7%, 1.7%, 5.8%, 13.3%, 6.4%, 5.9%, and 17.6%, respectively, and 5-year OS rates of 40%, 27%, 63%, 67%, 84%, 48%, and 82%, respectively 12. This novel genetic subtyping system was successfully applied in guiding personalized treatment for newly diagnosed DLBCL. In the study reported by WL Zhao et al, ibrutinib was added to R-CHOP for MCD and BN2 subtypes, lenalidomide for N1 and not otherwise specified (NOS) subtypes, decitabine for A53 subtype, tucidinostat for EZB subtype. This genetic subtype-guided R-CHOP plus X model (R-CHOP+X) was demonstrated to be superior over the classic R-CHOP regimen, and attained both higher CR rate and longer PFS 13.

Recently, Kotlov et al. reconstructed the lymphoma microenvironment (LME) of 4580 DLBCL patients using 25 functional gene expression signatures (F^GES^). They used an unsupervised community detection algorithm, which was used to analyze the correlation of F^GES^ among samples, resulting in four major LME clusters: 1) germinal center-like LMS (GC-like LMS), enriched in F^GES^ from cell types commonly found in germinal centers; 2) Mesenchymal subtype (MS-LME, abundant in F^GES^ from stromal cells and extracellular matrix pathways; 3) Inflammatory subtype(IN-LME), enriched in F^GES^ associated with inflammatory cells and pathways; and 4) Depleted subtype(DP-LME, was characterized by an overall lower abundance of microenvironment derived F^GES^). Microenvironment derived F^GES^ accounted for 15%, 33%, 25%, and 27% of the four subtypes, respectively. This LME-based categorization represents a new gene expression-based DLBCL subtyping and is to a degree related to the cell of origin (COO)-based approach. The IN-LME subtype contains a higher proportion of ABC-DLBCLs, but GC-like and MS-LME are more abundant in GCB-DLBCLs. These four different subtypes have different prognoses. Overall, GC-like and MS-LME subtypes confer a better prognosis, with a 5-year survival rate of approximately 80%, while, IN-LME and DP-LME subtypes confer poor prognosis. Especially DP-LME has a 5-year patient survival (PS) rate of approximately 60% 14. These researches are displayed in Table 1.

## Genomics

High-grade B cell lymphoma with MYC and BCL2 and/or BCL6 translocations is defined as a new entity in the 2016 WHO classification 15. Mounting evidences have confirmed that MYC/BCL2 co-expression and double translocations are important determinants of prognosis in DLBCL patients 16. MYC rearrangements occur in 5%-10% of DLBCL 17, half of which also have BCL2 rearrangements 18. Staiger et al. found that dual expression of MYC and BCL2 suggested a poor prognosis for DLBCL 19. The gene STAT3 located on human chromosome 17 regulates cell growth by upregulating MYC, a downstream target 20. Constitutive STAT3 activation is a distinctive feature of ABC subtypes in DLBCL. Huang et al. studied 185 DLBCL patients treated with R-CHOP and using IHC identified phosphotyrosine STAT3 (PY-STAT3) expression. Cell line-based siRNA assays also yielded an 11-gene PY-STAT3 activation signature, indicating that STAT3 activation is associated with poor survival in DLBCL R-CHOP treated with patients, particularly those with the ABC subtype 21.

Meta-analysis of four studies by Ghesquieres et al. revealed that a two-single nucleotide polymorphism (SNP) risk score can highly predict event free survival (EFS) (*p* =1.78×10^-12^) and is independent of treatment, International Prognostic Index (IPI), and cell source classification 22. They genotyped SNP loci, and used a log-additive genetic model with adjustment for age, sex and an age-adjusted IPI to calculate the hazard ratios (HRs) of EFS and OS in the sample and 95% confidence interval (CI). They found that the trait locus markers of EFS are 5q23.2 rs7712513 (close to SNX2 and SNCAIP genes; HR 1.39, 95% CI 1.23-1.57; *P*= 2.08×10^-7^) and 6q21 rs7765004 (close to MARCKS and HDAC2 genes; HR 1.38, 95% CI 1.22 - 1.57; *P* =7.09×10^-7^); the loci rs7712513 (HR 1.49, 95% CI 1.29 - 1.72; *P* =3.53×10^-8^) and rs7765004 (HR 1.47, 95% CI 1.27 - 1.71; *P* =5.36 × 10^-7^) were related to the patient's OS.

Using NGS technology, Pasqualucci et al. sequenced the whole-exome of 115 DLBCL samples and found that presence of multiple lesions targeting histone/chromatin modifying genes is a significant feature of the DLBCL tumor cell genome. Among them, mixed-lineage leukemia 2 (MLL2) is the most common mutation. In addition, they also found that β2-microglobulin (B2M) gene also has frequent mutations and deletions. Because the B2M gene is related to T cell immune recognition, its high frequency mutation or deletion makes DLBCL tumor cells insensitive to cytotoxic T cell-mediated killing, suggesting that tumors evade immune surveillance mainly via B2M deletion 23. Thus, B2M deletion may be a new prognostic indicator in DLBCL patients. Whole-exome sequencing and RNA sequencing (RNA-seq) of samples from 1,001 newly diagnosed DLBCL patients treated with rituximab-containing regimens, by Reddy et al., revealed 150 genetic drivers. Key oncogenes related to tumor cell growth (MYC, RHOA, SF3B1, MTOR, and BCL2) and genes related to tumor suppression (TP53, MGA, PTEN, and NCOR1) were identified. Knockout studies of the nine identified functional oncogenes showed that knockout of EBF1, IRF4, CARD11, MYD88, and IKBKB is selectively lethal in ABC DLBCL, while knockout ZBTB7A, XPO1, TGFBR2, and PTPN6 is selectively lethal in GCB DLBCL. Thus, they can be used as targets for the treatment of DLBCL patients and as biomarkers for predicting patient treatment outcomes. More importantly, 36% of DLBCL patients have genetic mutations in these nine drug targets and may thus respond well to chemotherapy. In addition, they also developed a genomic risk model and found that the 5-year survival rates of high-risk and low-risk patients in the complete remission group were approximately 60% and 90%, respectively, while the IPI was approximately 50% and 85%, respectively, indicating that the genomic risk model can make early prognostic predictions in DLBCL patients 24.

Circulating tumor DNA (ctDNA), DNA fragments released into the circulatory system by cancer cells, contain tumor-specific information. They are independent biomarkers used to assess the prognosis of DLBCL patients. ctDNA can be detected and quantified using NGS technology and allows noninvasive assessment of tumor kinetics 25, 26. Studies indicate a strong association between ctDNA and total metabolic tumor volume (TMTV) in DLBCL patients 27. Sidaway et al. studied 217 patients with DLBCL or primary mediastinal large B-cell lymphoma and found that ctDNA was measurable in 98% of the patients prior to treatment. These patients were subsequently divided into the discovery (n = 130) and validation (n = 73) groups. The dynamics of their ctDNA levels were monitored throughout treatment. ctDNA levels in responders reduced significantly within the first week of treatment. Thus, responders and non-responders could be identified at the end of the first treatment cycle. This finding was validated in the exploratory group: patients with early molecular response (EMR) and major molecular response (MMR), the 24-month EFS rate increased significantly, 83% vs. 50% (*P* = 0.0015) and 82% vs. 46% (*P* <0.001), and found that both EMR and MMR can predict the prognosis of DLBCL patients independently of the imaging results of positron emission computed tomography (PET-CT). These data indicate that the dynamic changes in ctDNA level can be early predictors of clinical prognosis in DLBCL patients, and ctDNA-based prognostic predictions do not depend on PET-CT imaging 28. In 2015, Roschewski et al. performed ctDNA quantitative analysis in a cohort of newly diagnosed DLBCL patients by monitoring serum ctDNA in a series of samples obtained during treatment and follow-up. They found that the rate of 5-year time to disease progression of patients with and without metaphase ctDNA was 41.7% (95% CI 22.2%-60.1%) and 80.2% (95% CI 69.6%-87.3%), respectively, and the OS rate was 65.4% (95% CI 42.4%-81.1%) and 83% (95% CI 73.1%-89.6%); the positive predictive value (PPV) of ctDNA was 63% and the negative predictive value (NPV) was 80%, with sensitivities and specificities of 47% and 88%, respectively. Therefore, ctDNA is better than PET-CT for predicting outcome and treatment response in DLBCL patients, suggesting that ctDNA may be used as predictive prognostic biomarkers in PET-CT-negative patients 29.

## Transcriptomics

Transcriptomic studies typically use RNA sequencing (RNA-seq), real-time quantitative PCR (qPCR), or microarray technology. A common application of transcriptomics in DLBCL research is to search for transcripts with altered expression in normal B lymphocytes, tumor cells, and different subtypes of tumor cells. In addition, non-coding RNAs, including microRNAs (miRNAs) and long non-coding RNAs (lncRNAs), also play important regulatory functions. Importantly, transcriptomic studies allow quantitative assessment of transcripts at various time points throughout the course of treatment, this information is essential for the prognostic assessment of DLBCL.

The biological basis of high-grade DLBCL with MYC, BCL2, and/or BCL6 rearrangements (HGBL-DH/TH-BCL2) was explored by Ennishi et al. They analyzed whole-exome sequencing, RNA-seq, and targeted resequencing and other data from patients with GCB-DLBCL (n = 157), including cases of HGBL-DH/TH-BCL2 (n = 25). They constructed a 104-gene (mRNA) double-hit signature (DHITsig) model for estimating OS rate using Kaplan-Meier survival analysis and found that the 5-year OS rate after R-CHOP treatment in DHITsig-negative (DHITsig-neg) and DHITsig-positive (DHITsig-pos) patients was 80% and 60%, respectively. Thus, they demonstrated the predictive utility of DHITsig on the prognostic outcome of DLBCL patients 30.

Recently, tumor microenvironment (TME) has been found to be critical in mediating immune evasion and treatment resistance in various cancers. Ciavarella et al. used the CIBERSORT algorithm to deconvolute gene expression profile (GEP) data from 482 untreated DLBCL patients and identified 45 tumor microenvironment (TME) genes of DLBCL. They quantified the expression of 45 tumor microenvironment (TME) genes in formalin-fixed, paraffin-embedded biopsy tissue samples using NanoString technique, and found that the 30-month OS rate was approximately 90% and 62.5% for those with high and low expression of all genes in TME, respectively (*P* =0.00017), and progression free survival (PFS) rate was approximately 75% and 60%, respectively (*P* =0.0069); COO/TME combination model predicted a 30-month OS rate of <25% and >75% in the high-risk and low-risk groups, respectively (*P* =0.00022). Thus, they concluded that TME has good prognostic risk stratification ability and its predictive power is significantly improved when combined with COO 31.

miRNAs can be used as prognostic biomarker of DLBCL. Sun et al. used miRNA PCR arrays to analyze the miRNA expression profiles DLBCL patients at three time points of treatment (372 serum samples from 20 patients), viz. diagnosis, remission, and relapse. They identified and used four miRNAs, i.e., miR21, miR130b, miR155, and miR28 to establish a 4-loop miRNA prognostic model. Multivariate analysis showed that the 4-loop miRNA prognostic model was significantly associated with poorer PFS and OS in both the training and validation cohorts 32. Unlike some investigators who focused mainly on collecting pre-treatment specimens, Bouvy et al. collected 68 miRNA samples from the sera of 19 DLBCL patients during treatment and analyzed them using microarray and qPCR techniques. They found that the plasma levels of miR-197, miR-20a, miR-451, miR-122, miR-19b, and miR-21 were closely associated with the sensitivity of DLBCL patients to chemotherapy, reflecting their prognostic utility in DLBCL patients. However, this preliminary study needs to be validated using a larger sample size 33.

In addition to miRNAs, lncRNAs have also shown important prognostic assessment value. Zhuo et al. studied three cohorts, including GSE31312 (n = 426), GSE10846 (n = 350), and GSE4475 (n = 129) from the Gene Expression Omnibus (GEO) database. Using differential expression analyses and weighted voting algorithm, they found that SubSigLnc-17, an lncRNA recognition marker is capable of distinguishing between GCB and ABC subtypes, with a sensitivity of 92.5%. SubSigLnc-17 was also identified for prognostic prediction 34. Although non-coding RNAs have predictive significance for DLBCL prognosis, the clinical application of miRNAs or lncRNAs as prognostic biomarkers for DLBCL is still relatively rare.

The main challenge in identifying biomarkers with transcriptomics, is the requirement of large number sample size, making it expensive. In future, the biomarker study will continue to be influenced by the rapid developments in transcriptomics albeit with newer unknown challenges. In addition, transcriptomics is not useful in cases where the disease is not caused by alterations in RNA sequences. Proteomics may be suited to study biomarkers in such cases.

## Proteomics

Proteomics encompasses identification of proteins and their posttranslational modifications, functions, and protein-protein interactions using two-dimensional chromatography (2D-LC) and two-dimensional liquid chromatography/tandem mass spectrometry (LC-MS/MS). Clinically, proteins or peptides expressed in tumor cells and body fluids of DLBCL patients have the potential to be prognostic DLBCL biomarkers. Recent developments in proteomics techniques, including increased sensitivity of detection has helped immensely in the discovery of novel prognostic biomarkers.

Some DLBCL tumor cells express programmed death receptor-ligand 1 (PD-L1) and its receptor the programmed death receptor 1 (PD-1) is expressed on T lymphocytes surface. The PD-1/PD-L1 pathway mediates immune escape from tumors. Therapies targeting the PD-1/PD-L1 pathway are clinically effective in treating Non-Hodgkin's Lymphoma (NHL) 35, 36. Kiyasu et al. suggested that while positive surface-expression of PD-L1 in DLBCL tumor cells is an independent prognostic factor for OS, the expression of PD-L1 in microenvironmental cells is not correlated with OS 37. DLBCL cells aberrantly express oncogenic transcription factor forkhead box protein 1 (FOXP1). Flori et al. found that DLBCL cells with aberrantly high FOXP1 expression had reduced expression of surface sphingosine-1-phosphate receptor 2 (S1PR2) 38. Since S1PR2 is required for the maintenance of GCB cell homeostasis 39, using an additional publicly available 2-gene expression profile (GEP) dataset they found that S1PR2 showed a near perfect negative correlation with FOXP1. Thus, suggesting that low S1PR2 expression, especially in combination with high FOXP1 expression, is an important predictor of poor prognosis in DLBCL patients 38. Certain leukocyte antigens (CD) expressed on B-lymphocyte membranes can be used as prognostic markers in DLBCL. Niitsu et al. in a cohort of 930 DLBCL patients found that the 5-year OS rate was 55% and 65% for CD5+ DLBCL (n = 102) and CD5- DLBCL (n = 828), respectively, while the 5-year PFS rates were 52% and 61%, respectively. The addition of rituximab to chemotherapy in patients with CD5+ DLBCL revealed a significant improvement in PFS (47.4% vs. 62.5%) at 4 years, but not in OS (57.8% vs. 63.5%) at 4 years 40. Therefore, they concluded that CD5+ could be a predictive biomarker of poor prognosis and response to rituximab therapy in DLBCL. Xu-Monette et al., found that CD37- DLBCL had significantly worse OS and PFS and significantly lower survival rate compared to CD37+ DLBCL patients after R-CHOP treatment. Thus suggesting that CD37+ is predictive of a benign prognosis in DLBCL patients 41. Meriranta et al. found that low Kelch-like protein 6 (KLHL6) expression predicted poor prognosis in DLBCL patients 42.

Maurer et al. using the FREELITE assay 43 found that 32% and 14% of patients (total n = 219) had elevated pretreatment serum free light chain (sFLC) or κ:λ FLC abnormalities; DLBCL patients with elevated sFLC had poorer EFS and OS compared to patients with normal sFLC. Therefore, they hypothesized that elevated sFLC was a stronger predictor of poor prognosis in patients with DLBCL 44. Witzig et al. conducted a 6-year follow-up study monitoring FLC concentrations and found that DLBCL patients fall into FLC monoclonal and polyclonal groups (n = 276 untreated) (monoclonal group EFS: HR 3.56, 95% CI 1.88-6.76, *P* < 0.0001; polyclonal group EFS: HR 2.56, 95% CI 1.50-4.38, *P* = 0.0006), suggesting that elevated FLC is a poor prognostic factor in DLBCL patients and may provide a new target for the treatment of DLBCL patients 45.

Proteomics has enabled a deeper understanding of the aberrant molecular alterations in DLBCL and has the potential to identify new prognostic markers and establish new molecular typing methods. Data on serum FLC in DLBCL patients are limited. Large sample size studies need to be performed and validated to enable faster clinical application. However, there are still some limitations and challenges with proteomics. First, large size of proteomics data makes their storage and processing into biologically meaningful results tough. Second, structural aberrations in protein and very low abundance may cause protein signals to be missed especially when using mass spectrometry (MS).

## Epigenetics

Epigenetics refers to study of heritable changes in gene expression such as histone modifications, DNA methylation, chromatin accessibility and long-range chromatin interactions 46, and chemical modifications of DNA and histones and the conformation of DNA 47 in nucleus determine epigenetics 48.

DNA methylation regulates gene expression, developmental processes and diseases 49. Intra-tumor methylation heterogeneity in DLBCL patients can predict the time to recurrence 50. Cytidine deaminase (AICDA) promotes B-cell demethylation in germinal centers. Teater et al. suggested that AICDA overexpression may be a biomarker of poorer prognostic outcomes in DLBCL patients. They found that DLBCL with high AICDA expression had higher intra-tumor methylation heterogeneity and cytosine methylation heterogeneity in tumor cells. The increased cytosine methylation heterogeneity was also associated with poor clinical prognosis in DLBCL patients 51.

N6-methyladenosine (m6A) is the most abundant and prevalent internal co-transcriptional modification in eukaryotic mRNAs. m6A methylation modification plays an important role in cancer development 52. Han et al. explored the function of m6A methylation modification in DLBCL and found that the high expression of m6A RNA regulatory gene PIWI-interacting RNA 30473 (piRNA) increased the level of m6A, resulting in poor prognosis in DLBCL patients. Thus, m6A methylation may be used for prognostic stratification in DLBCL 53.

Loss of CD20 is a major obstacle to R-CHOP treatment of relapsed/refractory DLBCL; histone deacetylation in DLBCL patients inhibits CD20 expression. Guan et al. demonstrated for the first time that *in vivo* and *in vitro* inhibition of histone deacetylase by chidamide significantly strengthened the tumor inhibitory effect of rituximab leading to improved prognosis in DLBCL patients. Thus, they established a synergistic role for Chidamide in the rituximab-R-CHOP treatment of DLBCL 54. Thus, it can be noted that histone deacetylation is one of the indicators of poor prognosis in DLBCL patients.

## Metabonomics

The metabolome includes all the low molecular weight (50-1500 Da) metabolites of an organism or cell. Metabonomics is a new discipline of qualitative and quantitative analysis of the metabolome to determine the relative relationship between metabolites and pathophysiological changes in diseases. In recent years, the rapid development of metabolomics has led to its increasing use in the analysis of altered metabolites in DLBCL tumor cells, and can be combined with other histological techniques to better identify prognostic biomarkers in DLBCL patients.

Using tumor cell gene expression as basis and consensus clustering Monti et al. identified three biological isoforms of DLBCL: 1) oxidative phosphorylation (Oxphos), 2) B-cell receptor/proliferation, and 3) host response (HR) 55. Oxphos -DLBCL have a predominantly glycolytic energy metabolism 56 enriched for genes involved in oxidative phosphorylation, mitochondrial function and electron transport chain. Patients with the 3 subtypes had similar 5-year survival rates (OxPhos, 53%; BCR/proliferation, 60%; and HR 54%; *P* =0.53) 55 suggesting that there may be metabolic heterogeneity in DLBCL and that this heterogeneity is genome-related. Ceriani et al. examined 103 DLBCL patients with primary mediastinal (thymic) large B-cell lymphoma (PMBCL) using (18) F-fluorodeoxyglucose(18FDG) positron emission tomography/computed tomography (PET/CT). They measured baseline 18F-FDG PET/CT by maximum standardized uptake value (SUVmax), metabolic tumor volume (MTV), and total lesion glycolytic (TLG) defined metabolic activity. All patients received combination chemotherapy based on adriamycin and rituximab; 93 received consolidation radiotherapy with a median follow-up of 36 months, at which time the OS rate was 100% in patients with low TLG and 80% in patients with high TLG (*P* = 0.0001), while PFS was 99% and 64%, respectively (*P* < 0.0001), leading to speculation that elevated TLG may be significantly associated with poorer PFS and OS in PMBCL patients, proposing that TLG on baseline PET appears to be a strong predictor of PMBCL outcome 57.

A common metabolic change found in tumor cells is elevated levels of glycolysis 58, and glyceraldehyde-3-phosphate dehydrogenase (GAPDH) is a key enzyme involved in glycolysis. Through unbiased analysis, Chiche et al. determined that GAPDH is the only glycolytic enzyme that can predict OS in DLBCL patients treated with R-CHOP. Based on GAPDH automated immunohistochemical (IHC) staining, a GAPDH scoring system was established. They quantified GAPDH expression levels in paraffin-embedded tissue microarray (TMA) of 43 newly diagnosed DLBCL patients and found that high GAPDH expression remained an important marker for predicting improved OS. Multivariate analysis (HR 0.603, *P* = 0.0371) showed that high GAPDH expression was an independent predictive biomarker for good prognosis in DLBCL patients 59. Alpha-ketoglutarate (α-KG) is a key metabolite in energy generation via the tricarboxylic acid cycle (TCA cycle) and can be generated during the catabolism of glutamine (Gln) to glutamate (Glu), a reaction catalyzed by aspartate transaminase (GOT1/2). By comparing the expression of GOT2 in normal B cells and different B-cell lymphomas, Feist et al. determined that abnormal GOT2 expression was characteristic of a subgroup of DLBCL. They then studied DLBCL patients (n = 157) treated with R-CHOP by including GOT2 expression in a Cox proportional risk model and found high GOT2 expression (HR 2.28, *P* = 0.03756) was significantly associated with shorter OS in DLBCL patients and could be used as a marker of poor prognosis in DLBCL 60.

Tome et al. (2005) determined that a redox signature score predicted poor prognosis in DLBCL patients, which incorporated features such as vitamin-D3 upregulated protein 1(VDUP1/TIP/TBP2), MnSOD, ZnSOD, EcSOD, thioredoxin reductase 1 and 2, catalase, thioredoxin, and microsomal GST. Patients with DLBCL were divided into four groups according to the quartiles of the oxidation reduction characteristics score, and their 5-year survival rates were compared: patients in quartiles 1 and 2 had similar 5-year survival rates (57%), patients in quartile 3 had lower survival rates (47%), and patients in quartile 4 had even lower survival rates (37%). Patients in quartile 4 had significantly shorter survival than those in quartile 1 (*P* <0.001) and quartile 2 (*P* =0.005). Thus, they hypothesized that the redox environment may play a role in the prognosis of patients with DLBCL 61. Kobayashi et al. studied natural killer cells (NK cells) and found that increased lipid metabolism in DLBCL patients produced fatty acids that effectively inhibited NK cell effects and cellular metabolism 62.

Metabolomics makes up for the shortcomings of genomics and proteomics. Its advantages are mainly reflected in the following aspects: A. even tiny genetic mutations have obvious metabolite changes; B. the metabolites are fewer in number than genes and proteins, and the study is more comprehensive; C. metabolite changes can directly reflect the pathological state of the organism. With the increasing use of metabolomics in clinical practice and the large number of samples being tested to build up a metabolic profile library, we believe that the study of metabolomic changes in DLBCL patients will become an effective method for identifying prognostic biomarkers in the future.

## Single-cell technology

### Single-cell sequencing

Single-cell sequencing is a new technique of analyzing genome, transcriptome, proteome, and epigenome at the single-cell level using high-throughput sequencing methods 63.

Tumors are composed of multiple cell types, including malignant cells, immune cells, and stromal cell subpopulations. The approach of using bulk tissue multi-omics to holistically understand tumors tends to obscure heterogeneity inherent in tumor cells 64. In contrast to conventional bulk sequencing, single-cell sequencing can accurately identify heterogeneous cell populations in tumors 65, especially in highly heterogeneous malignancies such as DLBCL.

### Single-cell transcriptomics

Over the past decade, with the development of NGS technology, single-cell RNA sequencing (scRNA-seq) has gradually become an important tool for studying differential gene expression within tumor cells at the transcriptome level 66. Autologous chimeric antigen receptor (CAR) T-cell therapy targeting CD19 shows good efficacy in patients with DLBCL. Deng et al. infused autologous axicabtagene ciloleucel (axi-cel) anti-CD19 CAR T-cell product in 24 patients with LBCL (2 PMBCL, 6 transformed follicular lymphoma (TFL), and 16 DLBCL), and then selected 137,326 residual cells for the whole transcriptome scRNA-seq test. They carried out a 3 month follow up study on these patients using PET/CT and found that after treatment, 50% patients experienced progressive disease (PD), 4% had partial remission (PR), and 38% had complete remission (CR). By analyzing the cell types in the infusion products of CR patients and PR/PD patients, they found a significant enrichment of depleted CD8 and CD4 T cells in the infusion products of PR/PD patients and a significant enrichment of memory CD8 T-cells and three times higher in the infusion products of CR patients than in PR/PD patients. Therefore, they analyzed the differentially expressed genes of CD8 T cells in CR patients and PR/PD patients and found that genes for basic leucine zipper ATF-like transcription factor (BATF), DNA binding inhibitor 2 (ID2), interferon gamma (IFNγ), effector molecules (GZMA, GZMB, and GNLY), and major histocompatibility class II (MHCII) molecules were associated with CD8 T cell depletion. They could serve as genetic markers of CD8 T-cell failure and could also predict the poor prognosis of patients with LBCL under this treatment. In addition, the investigators also measured the fold change of ctDNA alleles relative to somatic mutations in the plasma of patients on day 7 of treatment as early molecular response (EMR) and found that it was significantly correlated with clinical response (*P* = 0.008). Therefore, they suggested that the molecular response on day 7 after CAR T-cell therapy in DLBCL patients may serve as an early predictor of CAR T-cell efficacy markers 67. Their study provides a novel perspective in the search for molecular biomarkers in patients with LBCL undergoing cellular therapy.

ScRNA-seq emphasizes the importance of intercellular heterogeneity in health and disease phenotypic variability. It has been applied to discover new cell types, explore dynamic developmental processes, identify gene regulatory mechanisms, and reveal random allele expression. Efforts are being made to explore new transcriptome contents, such as spatial transcriptome with the combination of system function analysis and scRNA-seq**;** however, there is a critical limitation: scRNA-seq can only analyze the RNA profile of each cell once, and the RNA capture efficiency is not completely stable 68, 69. Therefore, improving scRNA-seq technology is crucial for the development of single-cell transcriptomes. Currently, single-cell transcriptomics are not well studied in DLBCL. Although there are many challenges, we believe that single-cell transcriptomics will play an important role in the search for prognostic markers in DLBCL.

### Other single-cell technologies

Despite the rapid development of single-cell genomic, single-cell epigenomic, and single-cell proteomic technologies 47, 70-82, their application in DLBCL has not progressed much. Obtaining high-quality and high-coverage genetic data of DNA molecules in single cells, protein concentrations with less noise (with slower protein degradation than RNA), as well as analyzing various single-cell histological data, remain major challenges that should be overcome 68, 69, 76, 83-85.

Single-cell genomics reveals genomic variability among single cells, transcriptomics, epigenomics and proteomics analyses study the functional state of single cells in an unbiased manner 86. Precision oncology has rapidly evolved, and proteogenomics is emerging as a new discipline in the clinic and is an important tool for achieving precision oncology therapy. Abundance of mRNA in the cell does not accurately reflect protein abundance, thus combining proteomics and genomics will be more useful for understanding the biological heterogeneity of cancer and identifying different prognostic outcomes in DLBCL patients 87.

## Radiomics

The concept of radiomics was first introduced by Dutch scholar Kumar in 2012 and has become a popular method of image analysis in recent years. Radiomics refers to the use of images obtained using computed tomography (CT), positron emission tomography (PET), or magnetic resonance imaging (MRI) data to extract representative features. Artificial intelligence methods such as machine learning or deep learning are used for quantitative analysis of these images and prediction of diseases. PET is one of the most commonly used imaging technologies for DLBCL diagnosis and treatment processes. The fusion of PET with CT or MRI can provide both functional metabolic and anatomical structural information of tumors, which has an important role in the diagnosis, clinical staging, efficacy prediction, and prognosis assessment of DLBCL.

At the 12th International Conference on Malignant Lymphoma, 2-Fluorine-18-Fluoro-2-deoxy-D-glucose-positron emission tomography/computerized tomography (^18^FDG-PET/CT) was formally included in the updated guidelines for lymphoma imaging for staging and prognostic assessment 88, 89. PET/CT is more accurate for DLBCL staging than CT alone, with upregulated staging being more common than downregulated staging; being highly sensitive to nodal and extra-nodal lesions, PET/CT can accurately assess the sites of these lesions (including skeletal involvement). Now, PET/CT has become a routine part of DLBCL staging and quantifying prognosis. The standard uptake value (SUV) is an indicator used to measure the tumor's ability to take up ^18^FDG. The maximum standard uptake value (SUVmax) of PET/CT at diagnosis was significantly associated with poor survival.

Interim PET/CT (iPET/CT) is an important auxiliary method for evaluating the efficacy and prognosis of DLBCL. The Deauville score and ΔSUVmax are two frequently used evaluation methods. Recently, in an iPET/CT evaluation study combined use of Deauville score and ΔSUVmax was proposed as an improved Deauville model for predicting the DLBCL response to chemotherapy 90. They retrospectively analyzed 593 patients who received R-CHOP treatment. They found that the 3-year PFS and OS rate of negative patients were 80.2% and 89.9%, respectively, with a complete response rate of 91.8%, while the 3-year PFS and OS rate of positive patients were only 12.5% and 27.3%, with a complete response rate of 29.2%, when evaluated by the improved Deauville method. Therefore, they concluded that the method could lead to early identification of chemotherapy-resistant patients, the timely adjustment of the treatment regimen, provision of alternative treatment, and prognostic improvements. However, iPET/CT prognostic assessment has not been standardized yet; further prospective studies are needed 91, 92.

Recent RICOVER-NORT cohort study showed that tumor volume ≥7.5 cm was a poor prognostic factor in elderly patients with aggressive lymphoma 93. In addition, Laetitia et al. found that total metabolic tumor volume (TMTV) measured at PET/CT baseline was a strong predictor of survival outcomes in patients with DLBCL. In patients treated with lenalidomide maintenance or placebo, elevated TMTV at baseline was significantly associated with poor PFS and OS. They concluded that baseline TMTV combined with parameters that respond to tumor load distribution could improve risk stratification at staging in patients with DLBCL 94.

**Figure 2:**
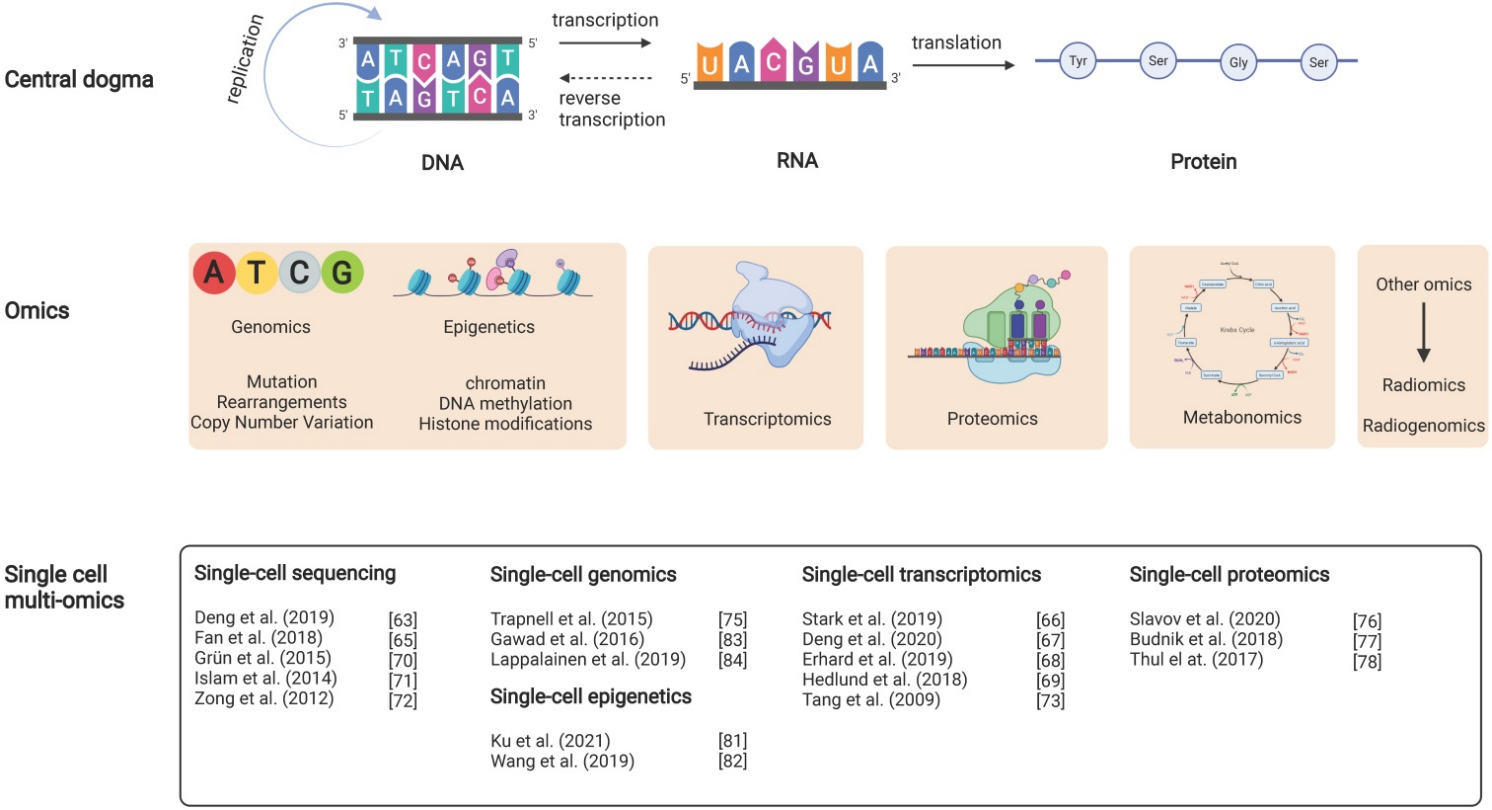
**Diagram of the relationship between the central dogma and DLBCL multi-omics studies.** It includes the interrelationship between the central dogma (layer 1), multi-omics studies (layer 2), and recent DLBCL studies in single-cell multi-omics (layer 3). For example, the DNA changed in the biological cycle in the central dogma corresponds with the possible mutations, rearrangements and copy number variants in DLBCL at the genetic level.

Lue et al. performed pre-treatment ^18^FDG PET/CT examinations on 83 patients pathologically diagnosed with DLBCL. They segmented ^18^FDG-avid lesions on PET images using the region-growing algorithm, and standardized lesions with a standardized uptake value (SUV) threshold higher than 2.5 were used for target delineation. Baseline ^18^FDG PET imaging omics feature, RLN_GLRLM_, was an independent prognostic factor. DLBLC tumors are more aggressive in patients with high RLN_GLRLM_ as compared to those with low RLN_GLRLM;_ they have a greater risk of recurrence/progression, and have a lower survival rate. The 5-year PFS and OS rate of patients with high RLN_GLRLM_ were 37.2% and 41.1%, respectively, while the 5-year PFS and OS of patients with low RLN_GLRLM_ were 91.7% (Figure 2) 95.

## Sampling sources in prognostic biomarker research of DLBCL

DLBLC biopsy samples can be sourced from various tissues and organs rich in prognostic biomarkers which include lymph nodes, spleen, bone marrow, blood, or cerebrospinal fluid (CSF). Lymphoma tissue is definitely the best source for biomarker research. However, several limitations exist. Increasing evidences have indicated the great heterogeneity of DLBCL, even intratumoral in a single patient, making it unsuitable for reflecting the genetic landscape from a single-site biopsy. Moreover, it is inconvenient to repeat tumor biopsy to determine the genetic changes during treatment. With the advent of liquid biopsy, samples from blood or CSF could be used in this scenario, and could highly match the findings from tumor tissues.

Blood samples are least invasive, cost-effective, and easily accessible. Blood biopsy samples can be studied at multiple levels, such as the genome, transcriptome, proteome, and metabolome. They can be an important source to discover prognostic DLBCL biomarkers. Peripheral blood from DLBCL patients can be subjected to liquid biopsy to detect circulating tumor cells (CTCs) and circulating cell-free tumor DNA (ctDNA or cfDNA). CTCs and ctDNA are of great interest for early detection of disease and assessment of prognosis, but their sensitivity and specificity still need to be improved 96. Rivas-Delgado A et al demonstrated in a population-based study that cfDNA could be an alternative source to assess the tumor burden and to show the tumor mutational profile and genetic classification, which have prognostic values and may guide future precision medicine in DLBCL 97. Frank MJ et al 98 showed in a multi-center study that monitoring of ctDNA could help detect early relapse after CAR-T cell therapy in relapsed/refractory DLBCL. Moreover, several biomarkers easily detected in blood samples could also be used to predict prognosis of DLBCL. sFLC has been shown to be a predictor of poor prognosis in patients with DLBCL 44, 45. Bittenbring et al. found that EFS of DLBCL patients can be predicted based on serum Vitamin-D levels. The 3-year EFS was 59% and 79%, for DLBCL patients with (≤8ng/ml) and without (>8ng/ml) Vitamin-D deficiency, respectively. Vitamin-D levels were also found to affect the OS response to rituximab treatment; the 3-year OS rates were 70% and 82%, respectively, for patients with and without Vitamin-D deficiency. In the multivariate analysis adjusted for IPI, the HR of EFS was 2.1 (*P* =0.008), and the HR of OS was 1.9 (*P* =0.040) and the prognosis of the two groups was significantly different. Treating for vitamin D deficiency (VDD) significantly increased rituximab-mediated cytotoxicity (RMCC) (*P* <0.001), suggesting that VDD is a predictor of poor prognosis in elderly DLBCL patients 99. In addition, Vaidya et al. proposed that absolute lymphocyte count (ALC) may also be a predictor of DLBCL prognosis in the era of R(X)-CHOP 100. In brief, given the non-invasive nature of liquid biopsy in peripheral blood and the rich resource of biomarkers in blood has not been fully exploited, it is believed that in future, blood will be a crucial source of sampling in the search for prognostic biomarkers in DLBCL.

Central nervous system (CNS) is a major site of DLBCL recurrence in many patients. CSF composition is altered in response to cancer. Lumbar puncture and CSF testing of patients can reveal that certain biomarkers have predictive significance for the prognosis of DLBCL patients. IL-10/IL-6 ratio, which is one of the indicators of poor prognosis of DLBCL, increased in the CSF of primary CNS lymphoma (PCNSL) patients. Kishimoto et al. also determined that TIM-1 is related to high expression of IL-10 101. Muñiz et al. used FCM to evaluate B cell-related markers in the CSF of patients with CNS lymphoma and suggested that higher levels of soluble CD19 (sCD19) were associated with CNS relapse rates in DLBCL patients, which are predictors of poor prognosis in DLBCL patients 102. Alvarez et al. found that elevated lactate dehydrogenase (LDH) levels were associated with an increased risk of CNS relapse 103. Cairo et al. also showed that elevated LDH levels were associated with an increased risk of CNS recurrence in adolescent (15-21 years) DLBCL patients 104. Moreover, ctDNA in the CSF plays an important role in the application of liquid biopsy in patients with CNS cancers 105. In the clinic, the diagnosis of CNS involvement in DLBCL patients is based on several clinical risk factors, including IPI, LDH, and the number of extra-nodal involvement (testis/adrenal gland/kidney) 106. A six-risk factor CNS-IPI model (five IPI factors with renal/adrenal gland involvement) developed by the German group for CNS diagnosis has been used in the clinic 107. In January 2021, Wang et al. published a new progress in the study of CSF-ctDNA in DLBCL patients. They found that increased free DNA (cfDNA) concentrations in the CSF of DLBCL patients correlated with high CNS-IPI. These findings underscore the significance of CSF-cfDNA in the detecting CNS tumors in DLBCL patients 108. Thus, CSF-cfDNA may be a putative prognostic biomarker in DLBCL patients.

## Conclusions

Predicting and determining the prognosis of patients with DLBCL is a long and complex research process. In the era of precision therapy, molecular typing of DLBCL using new technologies further enhances our understanding of the prognostic factors and biological behavior of DLBCL tumors. New molecular typing will play an important role in predicting the prognosis of DLBCL patients and will help to develop individualized treatment plans for patients and improve the cure and survival rates of DLBCL patients. In recent decades, research on DLBCL has advanced at multiple levels. The analysis of histological information and other databases has led to the discovery of multiple biomarkers that are predictive of therapeutic outcomes. Interestingly, single-cell histological studies of DLBCL are progressing rapidly, but have not yet been truly applied in the clinic. There is an urgent need to develop single-cell technologies in the future to promote novel biomarkers based on single-cell histological studies. Large scale clinical studies examining the presence of mutants, the impact of different biomarkers on the prognosis of treatment, the intricate network of signaling pathways within and between tumor cells, and whether traditional drugs and new targeted drugs can obtain reliable and clinically guided prognostic results based on new molecular DLBCL subtyping need to be conducted. In addition, studies on new research methods (aimed at reducing operational difficulty, cost, and time) with adequate sample size have become necessary for DLBCL research.

In summary, despite the many challenges of systems biology research, the integration of multi-omics studies continues to be a great boost to the identification of novel biomarkers of DLBCL.

## Figures and Tables

**Table 1:** Summary description of DLBCL subtyping and main research methods.

Year	Journal	Author	Methods	Subtype
**2000**	Nature	Alizadeh et al 5	DNA microarray analysis of gene expression	**·GCB:** GC B-like DLBCL **·ABC:** activated B-like DLBCL
**2002**	N Engl J Med	Rosenwald et al 6	DNA microarrays and analyzed for genomic abnormalities	**·GCB:** germinal-center B-cell-like **·ABC:** activated B-cell-like **·unclassified DLBCL: type 3 diffuse large-B-cell lymphoma**
**2004**	Blood	Hans et al 7	Immunohistochemistry	**·GCB:** 1)CD10(+) 2)bcl-6(+)& CD10+(+) 3)bcl-6(+),CD10(-)& MUM1(-) **·non-GCB:** 1)bcl-6(-)& CD10(+) 2)bcl-6(+),CD10(-)& MUM1(+)
**2018**	N Engl J Med	Schmitz et al 8	Exome and transcriptome sequencing, array-based DNA copy-number analysis, and targeted amplicon resequencing	**·MCD** (co-occurrence of MYD88L265P and CD79B mutations) **·BN2** (BCL6 fusions and NOTCH2 mutations) **·N1** (NOTCH1 mutations) **·EZB** (EZH2 mutations and BCL2 translocations)
**2018**	Nat Med	Chapuy et al 1	Comprehensive genetic analysis	**·Cluster 1** (BCL6 SVs, mutations of NOTCH2 signaling pathway ) **·Cluster 2** (biallelic inactivation of TP53, copy loss of 9p21.13/CDKN2A) **·Cluster 3** (BCL2 SVs and alterations of PTEN and epigenetic enzymes) **·Cluster 4** (distinct alterations in BCR/PI3K, JAK/STA T and BRAF pathway) **·Cluster 5** (18q gain, frequent mutations in CD79B and MYD88.)
**2020**	Blood	Stuart et al 9	Targeted sequencing	**·MYD88:** Strongly associated with ABC-type DLBCL, a poor prognosis **·BCL2:** Strongly associated with GCB-type DLBCL, generally favorable prognosis **·SOCS1/SGK1:** Predominantly GCB-type DLBCL, the most favorable prognosis **·TET2/SGK1:** A less strongly identifiable subtype, a favorable prognosis **·NOTCH2:** Not associated with any cell of origin, intermediate survival **·NEC:** A default category, intermediate survival
**2020**	Cancer Cell	Wright et al 10	LymphGen algorithm	**·MCD:** 5-year OS rate of 40% **·N1:** 5-year OS rate of 27% **·A53:** 5-year OS rate of 63% **·BN2:** 5-year OS rate of 67% **·ST2:** 5-year OS rate of 84% **·EZB-MYC+:** 5-year OS rate of 48% **·EZB-MYC-:** 5-year OS rate of 82%
**2021**	Cancer Discov	Kotlov et al 11	Transcriptomic analysis of the microenvironment	**·GC-like-LME:** commonly found in germinal centers **·MS-LME:** abundant in stromal cells and extracellular matrix pathways **·IN-LME:** associated with inflammatory cells and pathways **·DP-LME:** an overall lower presence of microenvironment-derived FGES

## Abbreviations

2D-LC: two-dimensional chromatography
ABC: activated B cells
ALC: absolute lymphocyte count
B2M: β2-microglobulin
BATF: basic leucine zipper ATF-like transcription factor
CAR: chimeric antigen receptor
CI: confidence interval
COO: cell of origin
CR: complete remission
CSF: cerebrospinal fluid
CNS: Central nervous system
CT: computed tomography
CTCs: circulating tumor cells
ctDNA: Circulating tumor DNA
DHITsig: double-hit signature
DLBCL: Diffuse large B cell lymphoma
EFS: event free survival
EMR: early molecular response
EMR: early molecular response
FOXP1: forkhead box protein 1
GAPDH: glyceraldehyde-3-phosphate dehydrogenase
GEP: gene expression profile
GCB: germinal center B cells
HRs: hazard ratios
IFNγ: interferon gamma
IHC: immunohistochemistry
IPI: International Prognostic Index
LME: lymphoma microenvironment
LC-MS/MS: two-dimensional liquid chromatography/tandem mass spectrometry
lncRNAs: long non-coding RNAs
m6A: N6-methyladenosine
miRNAs: microRNAs
MLL2: mixed-lineage leukemia 2
MMR: major molecular response
NEC: not elsewhere classified
NGS: next-generation sequencing technology
non-GCB: non-germinal center B cell-like
NPV: negative predictive value
NK cells: natural killer cells
OS: overall survival
Oxphos: oxidative phosphorylation
PCNSL: primary CNS lymphoma
PD: progressive disease
PD-1: programmed death receptor 1
PD-L1: programmed death receptor-ligand 1
PET-CT: positron emission computed tomography
PFS: progression free survival
PPV: positive predictive value
PR: partial remission
PS: patient survival
PY-STAT3: phosphotyrosine STAT3
qPCR: real-time quantitative PCR
R-CHOP: rituximab, cyclophosphamide, adriamycin, vincristine, and prednisone
RNA-seq: RNA sequencing
SCNAs: somatic copy number alterations
scRNA-seq: single-cell RNA sequencing
sFLC: serum free light chain
SNP: single nucleotide polymorphism
SUV: standard uptake value
SVs: structural variants
TMA: tissue microarray
TME: tumor microenvironment
TMTV: total metabolic tumor volume
VDD: vitamin D deficiency
